# Dimensions and temporal stability of medical students’ motivation: longitudinal evidence from a panel data survey

**DOI:** 10.1186/s12909-026-10249-z

**Published:** 2026-08-25

**Authors:** Stefanos A. Tsikas, Volkhard Fischer

**Affiliations:** Hannover Medical School, Dean of Studies Office – Academic Controlling, Hannover, Germany

**Keywords:** Study motivation, Intrinsic motivation, Extrinsic motivation, Medical studies, Study

## Abstract

**Background:**

Motivation is a key determinant of students’ engagement, learning strategies, and professional development in medical education. However, little is known about how different motivational dimensions change within individuals across training. The Hannover Screening of Study Motivation panel survey offers a unique opportunity to map how medical students’ motives evolve over time.

**Methods:**

Using the first five waves of data at Hannover Medical School, Germany (*N* = 1,218 subjects), we applied confirmatory factor analysis to validate three theoretically grounded dimensions of study motivation: extrinsic, autonomous, and prosocial. We then extracted standardized factor scores and modeled their trajectories with mixed-effects regressions.

**Results:**

Prosocial motivation (e.g., the desire to help others) dominates overall, but declines moderately but significantly within individuals. At the same time extrinsic motivation (e.g., job security, prestige) increases modestly. Autonomous motivation exhibits the greatest within-person stability. Motivational differences also align with career aspirations: students with stronger extrinsic motives gravitate toward high-status specialties, pro-social motivation is associated with social work and general patient care. Female students display less extrinsic and higher prosocial motivation, compared to males. We find no meaningful associations between any motivational dimension and early academic outcomes.

**Conclusions:**

The longitudinal patterns of study motivation suggest both pragmatic recalibrations, as students’ idealized views of medical practice meet clinical realities, and the subtle influence of hidden curricula that may erode altruistic motives. The moderate decline in prosocial motivation perhaps further reflects an incomplete professional identity formation process, highlighting the need for curricular and cultural strategies that sustain empathy and intrinsic engagement throughout medical training.

**Supplementary Information:**

The online version contains supplementary material available at 10.1186/s12909-026-10249-z.

## Introduction

### Background

Motivation is widely acknowledged as a critical driver of student engagement, learning, and professional development across higher education [1, 2]. In medical training, where cognitive demands, clinical responsibilities, and essential interpersonal skills converge, motivation underpins not only academic persistence but also the evolving professional identity of prospective physicians [3, 4]. Seminal research in Self-Determination Theory (SDT [5]), shows that autonomous motivation (intrinsic enjoyment and identified regulation) leads to deeper learning and greater well-being, whereas controlled motivation, driven by external rewards or pressures, encourages surface strategies and promotes exhaustion [6, 7].

Given rigorous selection procedures that assess not only cognitive ability but often also personal qualities and the desire to study medicine [8], motivation among medical students is generally assumed to be very high. Kusurkar et al. [9] argued that the perception of consistently high motivation partly stems from a longstanding focus on the “what” of learning rather than the “why”. Innovations such as problem-based learning [10, 11], spiral curricula [12], and competency-based medical education [13, 14] have begun to shift attention toward these motivational “why” questions. Indeed, Kusurkar et al. [15] have demonstrated that motivational profiles, i.e. clusters of intrinsic and extrinsic regulation, predict study effort, strategy use, and performance.

Yet evidence for motivation’s impact on measurable academic success in medical school remains mixed: while some studies link autonomous motivation to higher performance via deeper strategies [15], systematic reviews report overall inconclusive correlations between motivation and grades [16–18].

### Longitudinal perspectives on study motivation

Most investigations on study motivation to date are purely cross-sectional, offering valuable snapshots but are unable to distinguish between-person differences (who is more motivated) from within-person change (how each student’s motivation evolves over time). Longitudinal work by Hojat et al. [19–21] and Neumann et al. [22] has demonstrated group-level declines in empathy and motivation during clinical training, linking shifts to reduced professional commitment, empathic erosion, and variable academic outcomes. Similarly, studies by Kim and Jang [23], Mahoney et al. [24], Kool et al. [25], Kötter et al. [26], and da Silva Ezequiel et al. [27] report overall decreases in intrinsic motivation and partly increases in controlled regulation among medical students, while Quince et al. [28] and Blanco et al. [29] find no significant empathy declines but note gender differences.

However, these longitudinal analyses rely on mean-level comparisons across time points rather than tracking individuals’ trajectories. Thus, they cannot disaggregate whether observed changes reflect intra-individual shifts or merely compositional differences in the surveyed cohorts.

Panel data, by contrast, permit this very disaggregation. Fixed- and mixed effects models, applied to multi-wave panels, allow estimating both (a) stable, trait-like differences between students and (b) within-student fluctuations in motivation while eliminating all time-invariant confounders. To our knowledge, no study in medical education has combined psychometric validation of motivational dimensions with a multi-wave panel design to examine these dynamics.

The Hannover Screening of Study Motivation (HSM) aims precisely to fill this gap. Conducted annually at the Hannover Medical School (MHH) in Germany, the HSM connects items on study motivation and its dimensions with, for example, study progress, career preferences, and biographical data. With active student consent, the HSM can also be linked to administrative student statistics and exam records.

### Theoretical foundations

Within the HSM, we conceptualized three dimensions of study motivation – extrinsic, autonomous and prosocial – that map onto the aforementioned SDT and achievement goal theory [2, 30]: SDT locates motivation on a continuum from amotivation, through controlled extrinsic regulation (external, introjected), to autonomous regulation (identified, integrated, intrinsic). Crucial to this continuum is the process of internalization, which dictates how individuals adopt and integrate socially prescribed values or regulations into their self-concept. At the least autonomous end, external regulation is driven purely by external contingencies and separable rewards. Generally, but also in the context of medical education and medical professions, this manifests as pursuing a career for tangible, non-inherent outcomes such as high income, job security or social prestige. As motives become internalized, they shift through introjected and identified regulation, where a student recognizes the personal value and societal meaning of a goal (such as the desire to help others or social interactions), towards autonomous regulation. Here, motivation is fully integrated or inherently satisfying, characterized by genuine (academic) curiosity and a sense of self-actualization.

According to SDT, this developmental continuum is actively shaped by the frustration or satisfaction of three basic psychological needs, namely autonomy, competence and relatedness. Fulfillment of these needs fosters high-quality engagement and well-being, whereas controlled regulation often yields surface learning and exhaustion [6, 7]. Identified regulation bridges extrinsic and intrinsic motives, underpinning the prosocial dimension pivotal in medicine [15].

Achievement goal theory distinguishes mastery goals (focused on self-improvement) from performance goals (focused on normative comparisons). Mastery targets typically co-occur with autonomous regulation and predict adaptive strategies, whereas performance targets often align with controlled regulation and have mixed effects [30, 31].

### Study aims and contributions

Leveraging the currently five waves of the HSM, we address the following objectives:

1. Psychometric Validation: we apply confirmatory factor analysis (CFA) to validate three motivational dimensions (extrinsic, autonomous, and prosocial) within the HSM. Our aim is to provide a solid foundation for interpreting panel-derived motivational trajectories.
2. Within-Person Dynamics: we use standardized factor scores from the CFA to track students’ motivational trajectories over time, revealing differential stability across dimensions and potential ties to curricular experiences.
3. Substantive Links: we examine how the motivational dimensions relate to (a) career preferences, (b) study success (exam performance and study duration), and (c) discuss broader implications for professional identity formation and curriculum design.

By integrating theoretical considerations on study motivation, psychometric analysis, and a true panel design with mixed effects regression analyses, our study thus aims to move beyond cross-sectional assumptions to examine both intra- and inter-individual motivational dynamics in medical education.

## Data and methods

### The Hannover Screening of Study Motivation

The HSM has been conducted – albeit with occasional interruptions – at MHH for over 20 years. Its primary aim is to collect information on study motivation, academic progress, and career aspirations [32], complemented by biographical data such as educational background, work experience, place of origin, and parental education. Rather than serving as a psychometrically validated tool for measuring study motivation – such as the *Academic Motivation Scale* (AMS [7]) or the *Motivated Strategies for Learning Questionnaire* (MSLQ [33]) – the HSM was designed as a pragmatic instrument to support the university’s broader cycle of evaluation and quality assurance in education.

Closely related to the HSM is the annually conducted *Hannover Screening of Study Conditions* (HSC), which evaluates satisfaction with the curriculum, institutional infrastructure (e.g., lecture halls, learning spaces, cafeteria), and student support services [34]. Together, the HSM and HSC complement routine course evaluations by surveying attitudes and experiences that are not confounded by opinions on single teaching courses [35], and offer a more comprehensive perspective on the quality of medical education by helping identify areas for improvement [36].

Until 2020, the HSM was administered anonymously, using a self-generated personal code to link responses over time or across surveys (e.g., HSM and HSC). However, this code proved unreliable, and linkage to institutional data such as exam results was not feasible [37], limiting the (longitudinal) utility of the data. In 2021, the HSM was restructured as a true panel survey. Students are now invited to participate using a personalized identifier, and – subject to explicit consent – survey data can be linked to student records, including academic performance and study progression. Importantly, students decide in each year whether or not they wish to take part in a new wave. Participation is voluntary and not incentivized. The surveys also follow a modular structure: biographical characteristics are collected only at baseline, while later waves focus on updates, such as dissertation projects or comparisons to earlier study phases. This design reduces participant burden and increases efficiency by tailoring survey content to each respondent’s study stage.

Every year, 320 new medical students enroll at MHH. Each academic year begins with a new wave of the HSM and all students in the MHH model medical curriculum (Years 1–5), as well as those in the final clinical phase (Year 6), are invited to participate. Students who have completed at least one survey wave receive the shorter follow-up questionnaire.

Most items in the HSM use ordinal response formats or dichotomous yes/no questions, complemented by a few open-ended questions that allow students to elaborate on issues such as dropout considerations or perceived study delay. As shown in Table 1, the HSM covers a broad range of topics with comparatively brief and distinct item sets. This design was not chosen to achieve the psychometric precision of specialized instruments (for instance, in the domain of study motivation) but rather to establish a broad information base that supports the MHH’s evaluation and quality assurance goals. In developing the instrument, we explicitly accepted a trade-off between thematic breadth and methodological rigor: instead of maximizing internal consistency within single constructs, we prioritized capturing diverse aspects of the student experience while keeping the questionnaire concise. We also anticipated that this brevity and variation would encourage participation and reduce survey fatigue. In practice, the HSM achieves comparatively high response rates, exceeding those of both the HSC and the average course evaluations, despite the generally low participation in online surveys.

**Table 1:** Structure and contents of the HSM

Category	Content	# Items
General information	e.g. current living situation, commuting time	5
*High school information*	e.g. School subjects, committee work, internships	9
*Choice of study program*	Vocational training, previous studies, employment	7
Study motivation I	Desired study program, motivation to graduate, changes in study motivation	6
**Study motivation II**	Importance of various aspects in choosing the study program	9
Study motivation III	Opinions on study duration and importance of grades, thoughts on dropping out	5
Study progress	Doctoral project, internships, exchange programs	6
Assessment of competencies	Handling of literature, scientific methods, and medical findings	10
Self-assessment	‘Big-5’-style personality traits	10
Career choice	Opinions on careers in various fields of the medical profession	10
*Sociodemographic information*	Financing of studies, children, language skills, parental background	10

The indicated number of items does not include several free-text-fields scattered throughout the questionnaire. Categories in italics are (with the exception of single items) only included in the base questionnaire. The bolded category ‘Study motivation II’ is the focal point of the present study. Participants give their consent to using the data for quality assurance and research purposes at the end of the questionnaire. An English translation of the HSM can be found in section C of the Online Supplementary Materials

### Dimensions of study motivation

The HSM category *“Study Motivation II”* comprises the nine items that capture reasons for choosing to study medicine and pursuing a medical career. These items were designed to reflect prototypical motives, adapted to the specific context of medical education, and mapped onto the three theoretically grounded dimensions extrinsic, prosocial, and autonomous motivation.

Table 2 illustrates how each item embodies distinct strands of motivational theory, drawing on Self-Determination Theory (SDT), goal theory, and career orientation frameworks. Rather than aiming for comprehensive coverage, the nine items should be viewed as parsimonious yet theoretically anchored exemplars that capture the core motivational orientations most relevant to medical career choice. Conceptual frameworks guiding this process included the Academic Motivation Scale [7], Schein’s Career Orientations Inventory [38], and the SELLMO-ST [39], which addresses learning and performance motivation in higher education.

**Table 2:** Importance of various aspects in choosing the study program

Item name	Item description in the HSM	Motivational Dimension	Theoretical anchoring
Prestige	To get a prestigious job.	Extrinsic	External regulation, controlled motivation, performance-oriented goal
Social change	To contribute to social change.	Prosocial	Identified regulation, value orientation, communal goals
Job security	To obtain a secure professional position.	Extrinsic	Expectancy-value, risk reduction, controlled motivation
Helping others	To help other people.	Prosocial	Altruism, integrated regulation, empathy-driven behavior
High income	To achieve good income potential.	Extrinsic	External rewards, instrumental value, cost-benefit consideration
Self-actualization	Self-actualization.	Autonomous	Intrinsic motivation, personal growth, autonomy
Social contacts	To have a lot of contact with people.	Prosocial	Relatedness, belonging, social affiliation
Academic interests	To pursue subject-specific interests.	Autonomous	Intrinsic motivation, mastery goals, competence need satisfaction
Career options	To have many career opportunities.	Autonomous/ extrinsic	Identified regulation, future-oriented goals, self-determination continuum

**Fig. 1:**
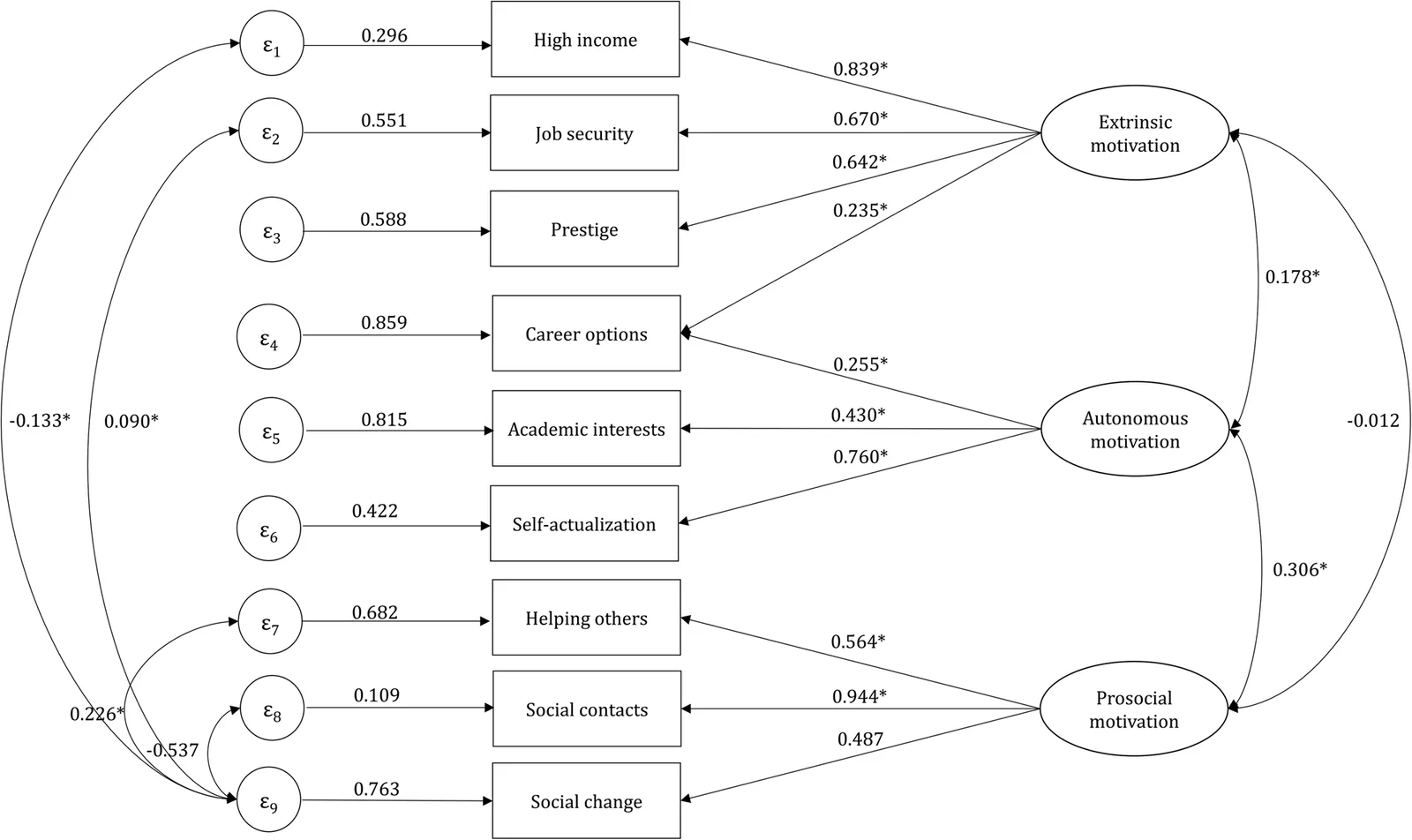
Results of the confirmatory factor analysis (CFA) with three latent factors. Notes: Structural equation model (SEM) with extrinsic, autonomous and prosocial motivation as latent factors. Coefficients have been standardized so that the latent factors = 1. Values represent correlations between indicators and the respective latent variable. Estimation method for the SEM was Full-Information Maximum Likelihood (FIML). Standard errors were adjusted on the subject-level for N = 1,218 subjects. The total number of observations is 2,193. Covariances between error terms were added based on modification indices (Lagrange multiplier tests) to improve model fit. See Table A in the Appendix for a complete SEM-output and Table 4 for goodness-of-fit statistics. *: *p* < 0.05

Table 2 lists the nine “Study motivation II” items, sorted according to their appearance in the HSM. Item scale: 1 = “no role” to 6 = “big role”. The “Item name” is used throughout this study; the “Item description” was presented to the participants on the questionnaire. The proposed motivational dimensions are validated with the confirmatory factor analysis (CFA) in Fig. 1 and Table A in the Appendix

A particularly instructive case in the HSM is the item *career options*, which exemplifies the inherent trade-offs in operationalizing motivation. While it resonates with autonomous motivation by emphasizing flexibility and self-directed development, it simultaneously reflects extrinsic considerations such as labor market value and professional security. In our SEM analysis, this hybrid nature is reflected empirically, with the item loading on both extrinsic and autonomous latent factors. This duality underscores the usefulness of confirmatory factor analysis in testing whether a deliberately condensed set of items can still capture the breadth of the proposed dimensions.

### Data

This study analyzes the first five waves (2021–2025) of the HSM panel. In total, up to 2,193 observations from 1,218 participants are available for analysis, contingent on participants’ consent. Around 46% of subjects contributed at least two observations, circa 25% participated in three or more waves. The primary reasons for attrition include voluntary non-participation and withdrawal from medical school, most commonly due to graduation or transfer to another university. Fewer than 2% of participants per wave declined to have their responses linked with administrative data and examination results. Students were contacted in two different ways: first-year students and those who had not responded to invitations in previous years received a link to the baseline survey. Students who had participated in one or several HSM waves received the follow-up questionnaire. The average response rate for the baseline survey was 18% and 42% in the follow-up waves.

The focal point in our analyses are the nine items presented in Table 2. These are complemented by ratings on different medical career paths, assessed on a scale from 1 (very reluctantly) to 6 (very much), and single items from the HSM categories “Study motivation I” and “Study motivation III”.

Given the panel data structure, sociodemographic characteristics, retrieved from the HSM and official student statistics, are only included in a supplementary analysis on academic performance, for which we use students’ results in the first part of the German medical licensing examination (M1 [40, 41]) as a proxy.

Within the HSM, an informed consent clause was incorporated, requiring participants to agree to the use of their responses and to the merger with student statistics and exam results. The informed consent and study regulations governing the use of personal data for evaluation/research and quality assurance purposes (in particular Sect.  14, para. 1–5 ‘*MHH Immatrikulationsordnung*’ and Sect.  17, para. 3 NHG (Higher Education Act in Lower Saxony, Germany)) rendered a separate approval by an ethics committee unnecessary. Participation and withdrawal in the questionnaire were voluntary at any given point. The research presented here is in accordance with the Helsinki Declaration.

### Empirical approach

Longitudinal panel data, where the same students are surveyed repeatedly over several years, contain two distinct types of information: between-person differences (how student A differs from student B) and within-person changes (how student A’s motivation fluctuates relative to their own baseline over time). Traditional statistical methods often used in medical education, such as cross-sectional correlations or group-mean comparisons, conflate these two levels.

To isolate within-person stability and shifts along between-person comparisons, we employ a two-step advanced approach: first, Confirmatory Factor Analysis (CFA) ensures that our motivational sub-scales measure the identical psychological concepts reliably across all time points. Second, Mixed-Effects linear regressions allow us to model individual trajectories while controlling for the fact that repeated measurements within the same students are naturally correlated.

As outlined, we begin our statistical analyses by testing the construct validity of our proposed three-factor structure through CFA within a structural equation modeling (SEM) framework, adhering to best practices in latent variable modeling [42, 43].

To maintain theoretical parsimony and because of the HSM’s nature described above, we specify a measurement structure where items, with the exception of the hybrid career options (see Table 2), are constrained to load exclusively on their primary assigned latent factor. While individual items like job security may conceptually border on identified regulation, we omitted exploratory cross-loadings for all items. Thus, we deliberately refrained from formulating various testable models that postulated correlations between different items. Instead, any shared variance and conceptual overlap between the motivational dimensions are captured through estimated covariances between the latent factors themselves.

To account for the panel structure of the data and repeated measures within individuals, standard errors in the SEM are clustered on the participant level, recognizing the non-independence of observations. Although more sophisticated approaches such as autoregressive cross-lagged panel models were considered, these were deemed infeasible due to the unfavorable ratio of latent constructs to available waves and observations [44]. Further details on the estimation of latent factor covariances, standard error computation, and a series of robustness and sensitivity checks are reported in the Results section.

From the SEM, we then estimate factor scores for all retained latent constructs and standardize them using *z*-scores. A positive factor score thus indicates a stronger endorsement of a specific motivational dimension, expressed in standard deviations relative to the full sample.

The standardized factor scores serve as dependent variables in mixed-effects (ME) panel regressions addressing two primary research aims. First, ME models account for both within- and between-subject variation by including random effects that capture subject-specific deviations from the sample mean. Second, we explore a range of time-varying predictors of motivational profiles, including the year of study, additional motivational items from the HSM, preferred career choices, and lagged factor scores from previous waves.

In the panel regressions, we do not include time-invariant characteristics such as sociodemographic variables and baseline academic achievement. This information is incorporated in a subsequent cross-sectional analysis to investigate associations between motivational profiles and academic performance.

Throughout the analysis, group differences and model coefficients are considered statistically significant if *p* < 0.05.

## Results

### Descriptive statistics

In our sample, 71% of participants are women, compared to ca. 65% of the total student body. Almost all students reported having the German citizenship. About one third of participants reported prior vocational training in a healthcare profession (see Table 3), reflecting the substantial weight clinical experience has in several admission quotas at MHH [41]. Overall, 82% of participants obtained their university entrance qualification (Abitur) from a general secondary school (Gymnasium), with the mean age at enrollment just under 22 years. Of the 1,218 participants, 920 had completed the first part of the medical licensing examination (M1). The mean M1-grade corresponds to a ‘B’ on an international scale.

**Table 3:** Summary statistics for variables used in this study

A: Sociodemographic information				
	%/Mean	*N*	SD	Median
Female (%)	71.03	1,216		
German (%)	98.68	1,216		
Gymnasium (%)	81.74	1,216		
Voc. Training (%)	34.41	1,203		
Age	21.64	1,216	3.46	20.33
M1-grade	1.84	920	0.64	2.00

**C: Other items on study motivation from the HSM questionnaire**				
	Mean	SD	Median	IQR
Preferred study program	5.30	0.89	5.50	1.00
Motivation to graduate	5.53	0.79	6.00	1.00
Certainty about graduation	5.41	0.84	6.00	1.00
Importance to graduate fast	4.19	1.46	4.33	2.00
Importance of excellent grades	3.55	1.27	4.00	1.83

**B: “Study motivation II” items**:				
	Mean	SD	Median	IQR
Helping others	5.34	0.84	5.67	1.00
Academic interests	5.30	0.81	5.50	1.00
Self-actualization	5.08	0.93	5.00	1.33
Career options	5.08	0.96	5.00	1.50
Job security	5.09	0.90	5.00	1.20
Social contacts	4.84	1.12	5.00	2.00
High income	4.62	1.02	5.00	1.00
Social change	4.54	1.14	5.00	1.20
Prestige	3.96	1.27	4.00	2.00

Summary statistics for sociodemographic information and HSM-items used in this study. All responses have been aggregated on the subject-level. SD: standard deviation; IQR: interquartile-range. Items in B) are sorted according to their mean value. Preferred study program: “How certain are you at the moment that you will complete your studies?” (1 = not at all certain to 6 = completely certain. Motivation to graduate: “How motivated are you currently to complete your studies?” (1 = not at all motivated to 6 = fully motivated). Certainty about graduation: “How confident are you that you will complete your studies?” (1 = not at all confident to 6 = completely confident). Importance to graduate fast and Importance of excellent grades: 1 = not at all important to 6 = very important

Panel B of Table 3 presents descriptive statistics for the motivational items from the “Study motivation II” section of the HSM. Ordered by mean scores (scale: 1 to 6), the results indicate a generally high level of agreement with the stated reasons for choosing to study medicine, accompanied by low variance, as evidenced by small standard deviations and interquartile ranges at the respondent level.

The desire to help others emerged as the most important motive, closely followed by academic and intellectual interests as well as aspirations for personal fulfillment. The high social prestige associated with the medical profession was rated lowest in importance. High income, frequent social interaction, and the potential to create positive societal change were also evaluated as important, albeit not as primary motivational factors.

Panel C of Table 3 reports summary statistics for additional HSM items on study motivation (item descriptions are in the table notes). Overall, students’ motivation levels can be described as very high, with only limited variance across individuals. Only the goal of achieving excellent grades played a comparatively smaller role. Divergent views were observed regarding the importance of graduating within the standard period of study, suggesting a trade-off between different rationales: on the one hand, the appeal of an earlier career start and financial independence; on the other hand, the desire to pursue a doctoral degree, gain international experience, or deepen academic expertise [16].

Longitudinal analyses across HSM waves and study years revealed largely stable motivational patterns. Approximately 14% of participants reported having considered withdrawing from medical school at some point, particularly during the first year. Reported reasons included feelings of being overwhelmed, high stress levels, and dissatisfaction, consistent with known risk factors for attrition in medical education [45, 46]. The actual dropout rate at MHH remains below 10% [40, 41].

### Confirmatory factor analysis

Figure 1 presents the structural equation model (SEM) estimated to validate the three-factor structure with three latent constructs – extrinsic, autonomous and prosocial motivation – each measured by their respective observed indicators. All coefficients in the SEM are standardized such that the variance of each latent factor is set to 1. The reported values represent standardized loadings, reflecting the strength of association between each indicator and its corresponding latent construct. Full Information Maximum Likelihood (FIML) estimation was employed. To account for the repeated-measures design and within-subject correlations, standard errors were clustered at the individual level (*N* = 1,218 students; 2,193 total observations across survey waves). The full output is presented in Table A in the Appendix.

Factor loadings ranged from 0.43 (academic interests) to 0.94 (social contacts), with *career options* standing out as an outlier (loadings < 0.30). One item, *social change*, did not reach statistical significance (*p* = 0.54), although its loading of 0.49 suggests that this result is more plausibly attributable to limited statistical power than to a lack of construct validity. Taken together, the pattern of factor loadings and their statistical significance generally supports the validity of the proposed three-factor structure. Nonetheless, we acknowledge that the hybrid nature of *career options* leads to weaker associations with the latent constructs, indicating that its contribution to the measurement model is more limited.

Across the three latent factors, factor loadings reveal clear differences in how strongly individual items capture the underlying constructs. For extrinsic motivation, most items align well, particularly *high income* (0.84) and *job securit*y (0.67), whereas the hybrid item career options contributes only marginally. Within autonomous motivation, *self-determination* (0.76) is a stronger indicator than *academic interests* (0.43), again with career options loading weakly. Prosocial motivation is most strongly represented by social contacts (0.94) and the desire to help others (0.56). Covariances between the latent constructs are consistent with theoretical expectations, showing low to moderate associations between autonomous and both prosocial and extrinsic motivation. In addition, error term covariances were specified where suggested by modification indices to improve the overall model fit.

The specified SEM achieved a very high coefficient of determination (CD) of 99.5%. Because clustering of standard errors at the individual level precluded the computation of additional goodness-of-fit indices, the model was also estimated without clustering; fit statistics are reported in column (1) of Table 4: the CD remained unchanged. The other indices indicate an overall acceptable model fit, with RMSEA = 0.063, CI: 0.054–0.071; pclose = 0.006) and CFI/TLI values above 0.90.

**Table 4:** Goodness-of-fit statistics

Fit statistic	(1) Full model	(2) Reduced model	Description
	(Fig. 1/Table A)	(Table S1)	
Likelihood ratio			
χ^2^_ms(19/13)	181.817	101.977	model vs. saturated
* p* > χ^2^	0.000	0.000	
χ^2^_bs(36/28)	3,780.878	3,472.976	baseline vs. saturated
* p* > χ^2^	0.000	0.000	
Population error			
RMSEA	0.063	0.056	Root mean squared error of approximation
90% CI, lower bound	0.054	0.045	
upper bound	0.071	0.066	
pclose	0.006	0.157	Probability RMSEA ≤ 0.05
Information criteria			
AIC	55,466.069	49,275.344	Akaike’s information criterion
BIC	55,665.325	49,451.828	Bayesian information criterion
Baseline comparison			
CFI	0.957	0.974	Comparative fit index
TLI	0.918	0.944	Tucker-Lewis index
Size of residuals			
CD	0.995	0.995	Coefficient of determination

Goodness-of-Fit (GoF) statistics for the SEM presented in (1) Fig. 1 and Table A and (2) for the reduced form without the item career options, presented in Table S1 (Online Attachment). For statistics beyond the CD, the models were estimated without clustering of standard errors on the subject-level. The SRMR is not presented because of missing values

In Table S1 of the Online Attachment, we report a reduced-form SEM excluding the item *career options* as a robustness check. The fit statistics are reported in column (2) of Table 4. Omitting this item slightly improves the overall model fit. Factor loadings for the three items reflecting extrinsic motivation remain robust. The factor loading for self-actualization increases, while the coefficient for academic interests becomes smaller. The factor loadings for the dimensions pro-social motivation change only marginally. Note that covariances between error terms, specified on the basis of modification indices (Lagrange Multiplier tests), were partly re-estimated due to the altered model structure and may have influenced the observed changes in factor loadings.

### Temporal stability of study motivation

**Fig. 2:**
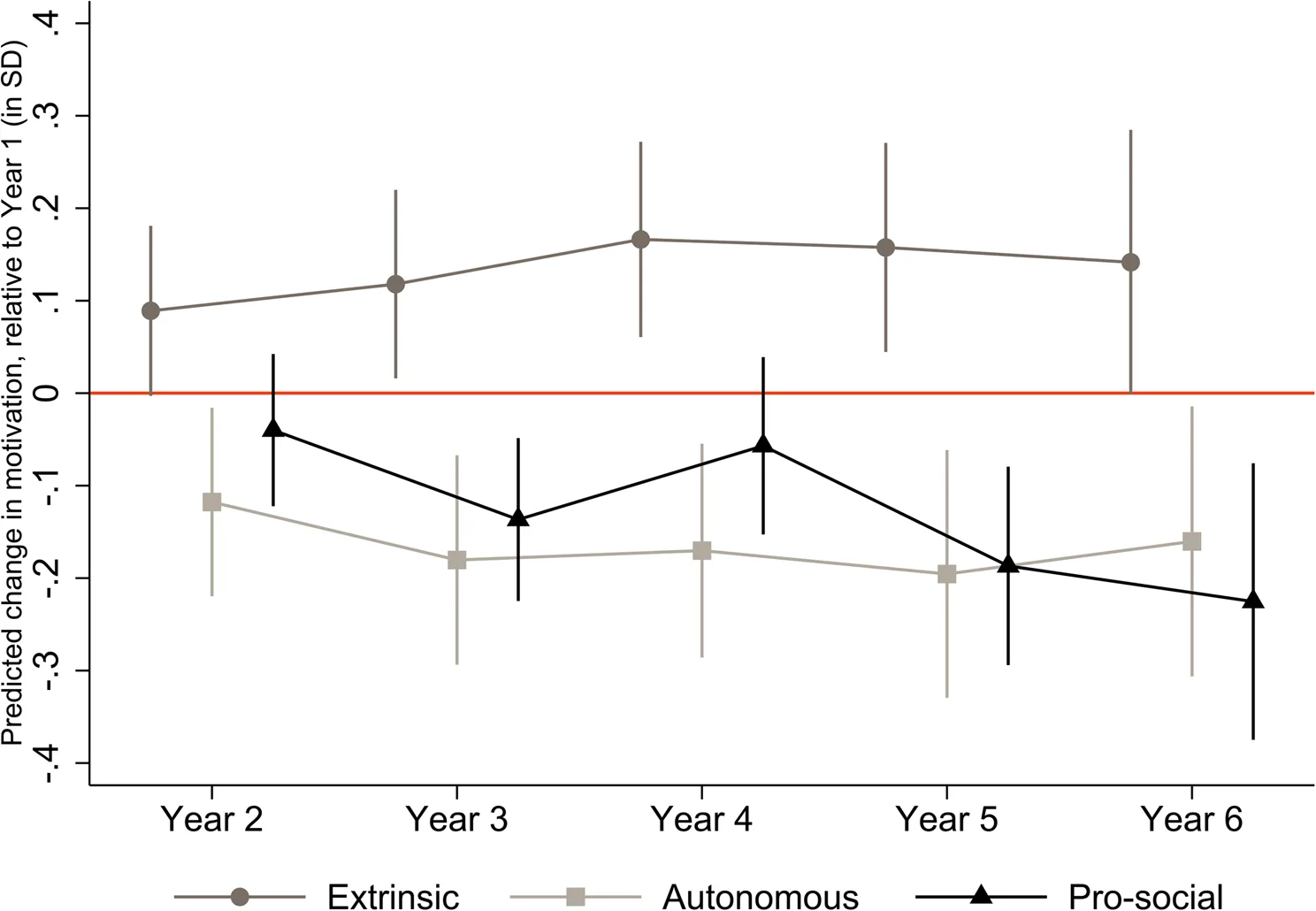
Changes in motivational dimensions within subjects over time. Notes: Predicted changes in extrinsic, autonomous and prosocial motivation over time. The estimates are partial effects of the respective factor score (z-score) retrieved from the SEM with respect to the study year, with year 1 serving as the baseline. Partial effects were estimated after a mixed-effects (ME) regression with the standardized factor score as the dependent variables and study-year dummies as the sole predictors. Standard errors in the ME-regression were clustered on the subject-level. Effects on the outcome variables are reported in standard deviations (SD). 95% confidence-intervals are indicated

Figure 2 illustrates the result of a mixed-effects (ME) panel regression using the standardized factor scores for each motivational dimension (estimated from the SEM-results) as outcome variables. The only predictor in the underlying model is a set of dummy variables for the year of study, with the first year serving as a reference category.

Extrinsic motivation, which is lowest in the first year, increases over the course of the study program. In years 2 through 5, extrinsic motivation is significantly higher than at baseline. However, the magnitude of these increases is modest, amounting to approximately 0.2 standard deviations.

Both autonomous and prosocial motivation are highest at study entry. Autonomous motivation remains quite stable across years 2–6, but is always significantly lower than at baseline. Prosocial motivation declines from year 1–3, and again after a slight uptick in year 4 (compared to the baseline). The drop is most pronounced towards the end of studies, but remains modest. Compared to the highest value in year one, pro-social motivation declines by approximately 0.25 SD.

### Other influences on motivational dimensions

Table 5 reports results from the ME-regressions that expand on the year-of-study specification by including additional predictors that may influence the validated motivational dimensions. Two criteria guided the selection of covariates: First, all variables needed to be time-varying (hence, sociodemographic characteristics were excluded). Second, the included variables had to exhibit at least small-to-moderate correlations (*r* ≥ 0.2–0.3) with the standardized factor scores for extrinsic, autonomous, or prosocial motivation. For example, personality traits (see Table 1) did not meet this threshold and were therefore omitted.

**Table 5:** Time-variant influences on the dimensions of study motivation

Dep. Variable: factor score…	Extrinsic		Autonomous		Prosocial	
	(1)	(2)	(3)	(4)	(5)	(6)
	ME	ME	ME	ME	ME	ME
Lagged dependent variable (t-1)		0.438*		0.404*		0.449*
		(0.023)		(0.025)		(0.018)
Factor scores (z-values):						
Extrinsic motivation			0.230*	0.186*	-0.029	0.001
			(0.026)	(0.034)	(0.023)	(0.028)
Autonomous motivation	0.246*	0.190*			0.323*	0.173*
	(0.026)	(0.028)			(0.023)	(0.027)
Prosocial motivation	-0.034	-0.034	0.360*	0.219*		
	(0.030)	(0.030)	(0.024)	(0.030)		
Preferred study program	-0.031	-0.072*	0.203*	0.137*	0.070*	0.058*
	(0.028)	(0.033)	(0.030)	(0.041)	(0.026)	(0.028)
Motivation to graduate	-0.006	0.004	0.062	0.077	-0.000	-0.032
	(0.029)	(0.037)	(0.033)	(0.041)	(0.028)	(0.029)
Certainty about graduation	0.082*	0.069	0.026	0.038	0.003	-0.024
	(0.032)	(0.045)	(0.030)	(0.045)	(0.027)	(0.034)
Importance of excellent grades	0.046*	-0.016	0.038*	0.048*	-0.003	-0.027
	(0.018)	(0.021)	(0.017)	(0.022)	(0.016)	(0.020)
Desired profession:						
General practice	0.026	-0.016	-0.046*	0.010	0.106*	0.060*
	(0.023)	(0.029)	(0.019)	(0.024)	(0.019)	(0.024)
Research & development	0.112*	0.078*	0.024	0.020	-0.143*	-0.129*
	(0.019)	(0.024)	(0.022)	(0.030)	(0.021)	(0.028)
University hospital	-0.064*	-0.026	0.096*	0.087*	-0.022	0.001
	(0.022)	(0.027)	(0.021)	(0.029)	(0.022)	(0.027)
Social work	-0.098*	-0.073*	0.002	0.000	0.116*	0.097*
	(0.021)	(0.029)	(0.020)	(0.026)	(0.020)	(0.028)
General hospital	0.038	0.046	-0.006	-0.009	0.060*	0.042
	(0.020)	(0.024)	(0.020)	(0.027)	(0.020)	(0.025)
Military career	-0.037	-0.045	-0.030	-0.057*	0.005	-0.015
	(0.020)	(0.027)	(0.021)	(0.029)	(0.019)	(0.024)
Study year FE	Yes	Yes	Yes	Yes	Yes	Yes
Observations	2,146	806	2,146	806	2,146	806
Number of subjects	1,205	473	1,205	473	1,205	473

Mixed effects (ME) regressions with the standardized factor scores retrieved from the confirmatory factor analysis (SEM) as dependent variables. Even-numbered models include only subjects who participated in at least two consecutive waves of the HSM. This is due to the inclusion of the lagged dependent variable, which measures the impact of the factor score in period *t-1* on extrinsic, autonomous, and prosocial motivation in period *t*. The explanatory variables ‘Desired profession: …’ are factor scores retrieved from an explanatory factor analysis (EFA) with the responses on the HSM question ‘In which area would you like to work later?’ The results of this EFA are presented in Table S2 in the Online Supplement. Standard errors, clustered on the subject-level, are in parentheses. Occasional non-responses or missing data in some variables can result in listwise deletions and slightly reduces the overall number observations

*: *p* < 0.05

Table 5 also includes correlations with the other motivational factor scores and an autoregressive term for the dependent variable in even-numbered models. This setup allows us to assess how motivation at time *t* is influenced by motivation at *t-1*. Since this requires two consecutive observations per subject, the sample size becomes smaller.

The results show that, across all three dimensions, prior motivation (*t-1*) is a strong and statistically significant predictor of current motivation (*t*). This underlines the relative stability of individual motivation over time (see Fig. 2). As in the SEM, weak correlations are found between extrinsic and autonomous motivation, as well as between autonomous and prosocial motivation. Overall, the lagged dependent variables as well as the smaller sample size in the even-numbered models does barely alter the relations between predictors and outcome variables.

Among the additional time-varying motivation-items, only the agreement that medicine is one’s desired field of study is weakly associated with the motivational dimensions: it is negatively correlated with extrinsic and positively associated with autonomous and pro-social motivation.

Regarding career preferences, several very weak but statistically significant associations emerge. Extrinsic motivation is positively associated with preferences for careers in research & development, and negatively with social work and a career as hospital residents. Higher autonomous motivation is associated with preferences for careers as a resident in a university-clinic. Prosocial motivation is positively related to a preference for private practice and social work/international development, and negatively associated with research & development.

Table 6 examines how academic success – operationalized as both the final grade in the first stage of medical studies (M1) and the duration of study until M1 – relates to students’ motivation. As these outcome variables are time-invariant, we applied a cross-sectional regression framework and included several sociodemographic control variables.

**Table 6:** Time-invariant influences on the dimensions of study motivation

	(1)	(2)	(3)	(4)	(5)	(6)
	Extrinsic	Autonomous	Prosocial	Extrinsic	Autonomous	Prosocial
M1-grade	0.007	-0.028	-0.017			
1 (‘A’)	(0.072)	(0.076)	(0.073)			
*Reference: 2 (‘B’)*						
3 (‘C’)	0.163	0.161	0.192*			
	(0.105)	(0.099)	(0.087)			
Study time (M1)				-0.032	0.018	0.021
				(0.033)	(0.029)	(0.031)
Female	-0.146*	-0.013	0.167*	-0.157*	-0.030	0.152*
	(0.072)	(0.075)	(0.069)	(0.073)	(0.074)	(0.070)
German	0.437	-0.125	0.173	0.367	-0.154	0.171
	(0.374)	(0.265)	(0.270)	(0.381)	(0.278)	(0.281)
Gymnasium	0.020	-0.148	-0.181*	0.008	-0.164	-0.241*
	(0.093)	(0.090)	(0.085)	(0.097)	(0.091)	(0.085)
Voc. Training	0.063	0.141	-0.036	0.064	0.122	-0.036
	(0.111)	(0.090)	(0.086)	(0.118)	(0.089)	(0.088)
Age	-0.029	-0.013	-0.007	-0.028	-0.010	-0.007
	(0.020)	(0.013)	(0.012)	(0.021)	(0.013)	(0.013)
Observations	919	919	919	888	888	888
R^2^	0.014	0.011	0.018	0.015	0.007	0.015

OLS regressions with the standardized factor scores retrieved from the confirmatory factor analysis (SEM) as dependent variables. The predictors of interest are the grade achieved in the first phase of the study program (M1) and the study time until M1 was achieved. M1 grades 4 (‘D’) and the failing grade 5 are not included due to very low numbers. Sociodemographic indicators (see Table 3) are included as control variables. Robust standard errors are in parentheses. Occasional non-responses or missing data in some variables can result in listwise deletions and slightly reduces the overall number observations

*: *p* < 0.05

Overall, we find very few associations between motivational constructs and academic success. Only lower-than-average grades are significantly associated with higher levels of prosocial motivation.

In addition, we observe that female students report higher prosocial motivation and lower extrinsic motivation than their male peers. Students who completed their secondary education at a Gymnasium (the most academically oriented track of the German school system) report lower intrinsic motivation (both autonomous and prosocial) compared to graduates of other school types.

A very low *R*^*2*^ further indicates that grades and sociodemographics are far less relevant for motivation compared to the path-dependency and time-variant variables included in Table 5.

## Discussion

### Summary

In this study, we leveraged the Hannover Screening of Study Motivation, an online panel survey of German medical students to examine the structure, stability, and predictors of motivational dimensions over time.

In line with our first research aim stated in the introduction, the structural equation model validated three motivational dimensions – extrinsic, autonomous and prosocial – anchored in motivation theory. Despite the limited number of items per construct, reflecting the overall design, thematic breadth and goals of the HSM, the measurement model achieved a solid overall fit. The three-factor structure proved robust, with most items loading clearly on their intended constructs. However, the hybrid item *career options* showed the weakest association with its factors, indicating that it perhaps could be replaced with an item more clearly aligned with e.g. autonomy or competence. Furthermore, while certain items like job security inherently carry psychological nuances that could transition from purely external rewards to a personally meaningful professional future (thus, identified regulation), our CFA structure purposefully refrained from specifying cross-loadings. A robustness check excluding the hybrid item career options yielded a slightly improved model fit, and the remaining items absorbed part of its variance without altering the validity of the main model.

Standardized factor scores retrieved from the SEM were then analyzed with mixed-effects regressions to pursue our second research aim, capturing both within-person change and between‐person differences. Overall, all motivational dimensions are temporally quite robust, although nuanced developments can be observed: extrinsic motivation increased modestly over time (≈ 0.2 SD) but remained substantially lower than intrinsic motivation throughout the study period. Pro-social motivations peaked at matriculation and gradually declined, showing the steepest erosion (≈ -0.25 SD) by the end of the program. Autonomous motivation declines after year 1, but remains stable afterwards. Prior motivation emerged as the strongest predictor of current motivation across all three dimensions.

Our third research aim was to analyze substantive links with motivational dimensions: career preferences and the importance attributed to medicine as one’s desired field also showed consistent and expected, but generally very weak associations. Our examination of links between motivational profiles and study success was limited to early outcomes, but corroborated the inconclusive evidence in the literature (see, e.g., the review by Kusurkar et al. [17]).

### Contributions to the literature

Our work advances the literature in three ways. First, by using panel data and mixed effects analyses, we overcome the limitations of cross-sectional designs that dominate studies of medical student motivation. This approach provides novel insights into the temporal dynamics of motivation – specifically, the gradual decline in prosocial (or altruistic) motivation – that have been so far demonstrated only with longitudinal mean-group comparisons (e.g [19, 21, 22]). Second, our study demonstrates a methodological combination that can elevate longitudinal research in medical education. By first using structural equation modeling to validate our motivational scales, we ensured that we were measuring clean, distinct dimensions of motivation without having to rely on a comprehensive, standardized inventory. By then feeding retrieved factor scores for each dimension into mixed-effects regressions, we were able to connect psychological measurement with true longitudinal tracking. In practical terms, this combination allowed us to simultaneously confirm that our questionnaire works reliably while tracking motivational trajectories over five waves, avoiding statistical distortions common in simpler analyses [42, 43]. Third, by linking motivational dimensions to career preferences and, in a separate cross‐sectional analysis, to academic performance, we contextualize motivation within students’ evolving professional identities [47, 48] and educational outcomes.

### Interpretation and relations to previous research

Our findings corroborate the overarching tenets of SDT, which posits distinct extrinsic and intrinsic (autonomous) motivators [49, 50]. The positive correlation between autonomous and prosocial motivation aligns with SDT’s notion of identified regulation and intrinsic goal pursuit.

In our study, we demonstrate that these relationships remain largely stable yet nuanced within subjects over time. For the observed decline in prosocial motivation, a plausible explanation is a “reality shock”: as students progress through clinical placements (e.g., practical courses, clerkships, and the practical year), they encounter the stress, routine tasks, documentation burdens, and sometimes impersonal or cynical attitudes of real-world practice, which clash with their initial idealistic views of the physician’s role: Hojat et al. [21] document a longitudinal erosion of empathy among third-year medical students, while Neumann et al. [22] show in their systematic review that empathy, and by extension prosocial motivation, is undermined by hidden curricula [51] such as hierarchical pressures, time constraints, emotional distancing, and poor role modeling.

Within our data, prosocial and autonomous motivations follow comparable trajectories, underscoring their conceptual kinship as facets of intrinsic motivation. However, autonomous motivation declines less sharply from its baseline value, a finding consistent with SDT’s assertion that autonomous motives, rooted in internal values and interests, are more resilient to external pressures [50, 52]. Vallerand et al. [7], using the AMS, similarly demonstrate that intrinsic motivation correlates strongly with stable personality traits and remains relatively constant over time.

Further, we observe a modest increase in extrinsic motivation where intrinsic motivation wanes; yet the latter continues to predominate. Interestingly, this slight longitudinal increase in extrinsic motives seemingly contradicts classic SDT predictions, which typically anticipate that students become more-self-determined over time as they successfully internalize the value of their studies. This divergence can be perhaps understood through the changing situational context of advanced medical training. As graduation and the transition into professional practice approach, concrete career and life planning considerations become more prominent. Students face cumulative academic pressures as well as emerging responsibilities such as financial obligations or family planning. Under these conditions, even highly intrinsically motivated students (which is typically the case [17, 53], also in our data) might be compelled to develop a heightened awareness of external contingencies. Endorsing job security or high income more strongly in later years does not necessarily imply a profound loss of altruism; rather, it reflects a pragmatic calibration as students prepare to enter the labor market.

This interpretation aligns with broader medical education literature showing that external regulation and a focus on structural outcomes often peak during clinical clerkships and final training phases. For instance, studies by Cho et al. [54] and da Silva et al. [55] found that extrinsic goal orientation and emphasis on rewards rise as students transition into the clinical sphere. Kusurkar et al. [15] further report that higher extrinsic motivation correlates positively with exhaustion levels, suggesting potential trade-offs between external incentives and well-being.

Further, our interpretation points to a deeper connection between motivation and *Professional Identity Formation* (PIF): motivation is both an individual attribute and part of the socialization process through which students internalize professional norms and roles [3]. When the hidden curriculum emphasizes efficiency, hierarchy, and detachment [56, 57], prosocial motives, or empathy, may erode alongside emerging professional self-concepts. Our modest decline of prosocial motivation thus may reflect an incompletely supported PIF process. Crucially, this downward trajectory should not be overinterpreted as an erosion of altruism: throughout all observed waves, prosocial motives consistently remain at a very high absolute level. The observed shifts therefore represent subtle individual recalibrations rather than a loss of core values.

The weak associations between dimensions of study motivation and career aspirations we find in our data also point in the direction of evolving professional identities: high pro-social motivation is linked to a preference for humanitarian work and primary care, autonomous motivation is associated with careers in university hospitals, e.g. as clinician scientists, and extrinsic motivation is linked to specialties with higher earnings or greater job security.

To conclude, we must stress that motivation is largely stable over time and dominated by intrinsic motives, albeit with context-driven adaptations in prosocial and extrinsic dimensions. Such adaptations appear pragmatic rather than alarming. Nevertheless, educators should actively leverage existing intrinsic motivation – particularly by fostering autonomy, competence, and relatedness [50] – and guard against the corrosive effects of hidden curricula. Educational strategies that integrate reflective practice, constructive feedback, mentorship, and interprofessional collaboration can sustain prosocial motives and support healthy PIF [3, 4].

### Limitations

The HSM is a multifaceted instrument that covers various domains beyond study motivation and includes only nine items to assess its different dimensions. While this brevity reflects a pragmatic design that can be applied for a variety of contexts, it may insufficiently capture nuanced motivational sub-dimensions. Furthermore, self-reported data are inherently susceptible to social desirability bias. It is possible that observed declines in prosocial motivation partly reflects a behavioral shift in response tendencies. Whereas first-year students might feel compelled to endorse idealized reasons for entering medicine, advanced students, as we have discussed, might express more pragmatic views.

Although our structural equation model confirms the underlying factor structure, the study is not without methodological constraints: Missing data and panel attrition, while addressed through full information maximum likelihood (FIML), inevitably reduce statistical power in later waves and may lead to wider confidence intervals, particularly for Year 5–6 estimates. Low participation rates are a well-known challenge in voluntary, non-incentivized online surveys [58]. Low participation and attrition between waves also prevented the use of autoregressive and cross-lagged SEM designs. Furthermore, our study is limited to a single institution and one medical degree program, which restricts the generalizability of our findings.

Our examination of academic success is cross-sectional and limited to M1 exam outcomes. Longitudinal tracking of clinical performance and professional development (e.g., workplace-based assessments or licensing examinations) would be valuable to draw stronger causal inferences. The lack of a robust link between motivation and academic success in our data may also be attributable to the highly selected nature of medical students, who are typically admitted based on cognitive excellence and thus form a relatively homogeneous group in terms of academic ability [59]. While the resulting weak or non-significant associations with exam outcomes might at first glance appear minor, they constitute an important empirical finding: they suggest that within highly competitive admission regimes, early academic performance is heavily restricted in variance, meaning that motivational orientation perhaps functions less as a performance differentiator and more as a driver of career path alignment.

## Conclusions

By applying a SEM-validated panel design to multi-wave data, this study provides novel empirical insights into the temporal dynamics of medical students’ motivation. Our findings demonstrate that while medical students maintain high levels of autonomous and intrinsic motivation throughout their training, their motivational profiles undergo nuanced, pragmatic recalibrations. Specifically, prosocial motives experience a moderate, within-person decline across clinical training years (though remaining at a high absolute level) while extrinsic considerations slightly increase as graduation approaches. While motivational dimensions display very limited predictive power for early academic outcomes, we conclude that they can function as an internal driver of thematic interest and career choice.

The importance and relevance of these findings for medical education are twofold: Methodologically, our work underscores the necessity of utilizing true panel data to distinguish genuine intra-individual trajectory shifts from compositional cohort differences. Practically, the observed shifts highlight the subtle influence of clinical environments and hidden curricula on professional identity formation. Rather than signaling a fundamental loss of altruism, these adaptations reflect a realistic calibration as students encounter clinical practice and prepare for professional entry. Medical faculties should leverage these insights by designing curricula that actively safeguard altruistic motives, e.g., through structured reflection, mentorship, and support for autonomy and relatedness, to sustain prosocial engagement alongside evolving professional responsibilities. 

## Supplementary Information

Supplementary Material 1.

Supplementary Material 2.

## Data Availability

The data analysed during this study is not publicly available due to its proprietary nature and privacy concerning administrative data used for this article. Data are available from the corresponding author upon reasonable request.

## Abbreviations

AMS: Academic Motivation Scale
CD: Coefficient of determination
CFA: Confirmatory Factor Analysis
CFI: Comparative fit index
CI: Confidence interval
FIML: Full information maximum likelihood
HSC: Hannover Screening of Study Conditions
HSM: Hannover Screening of Study Motivation
M1: First part of the German national medical licensing exam
ME: Mixed effects
MHH: Hannover Medical School
MSLQ: Motivated Strategies for Learning Questionnaire
PIF: Professional identity formation
RMSEA: Root mean standard error approximation
SD: Standard deviation
SEM: Structural Equation Model
SDT: Self-determination theory
TLI: Tucker-Lewis index
