# Supplementary material for: Dimensions and temporal stability of medical students’ motivation: longitudinal evidence from a panel data survey
Source: BMC Med Educ. 2026 Aug 25;26:1367. doi: 10.1186/s12909-026-10249-z (PMC13504741; doi:10.1186/s12909-026-10249-z)
Supplement: Supplementary file 1 [file 12909_2026_10249_MOESM2_ESM.pdf]

Supplementary Materials

A. Reduced-form CFA without the item 'career options'

Table S1: Structural Equation Model (SEM), reduced form without the item 'career options'

Factor	Coeff.	Robust SE	z	P>z	95%-confidence interval	
**Extrinsic motivation**						
High income	0.851	0.025	33.37	0.000	0.801	0.901
Job security	0.667	0.027	24.54	0.000	0.613	0.720
Prestige	0.655	0.025	25.98	0.000	0.606	0.705
**Autonomous motivation**						
Self-actualization	0.840	0.123	6.81	0.000	0.598	1.082
Academic interests	0.389	0.062	6.28	0.000	0.268	0.511
**Prosocial motivation**						
Helping others	0.610	0.061	10.05	0.000	0.491	0.729
Social contacts	0.867	0.080	10.81	0.000	0.710	1.025
Social change	0.352	0.047	7.47	0.000	0.260	0.445
var(ε.High income)	0.276	0.043			0.203	0.376
var(ε.Job security)	0.556	0.036			0.489	0.631
var(ε.Prestige)	0.571	0.033			0.509	0.639
var(ε.Self-actualizazion)	0.294	0.207			0.074	1.171
var(ε.Academic interests)	0.849	0.048			0.759	0.949
var(ε.Helping others)	0.628	0.074			0.499	0.791
var(ε.Social contacts)	0.248	0.139			0.082	0.746
var(ε.Social change)	0.876	0.033			0.813	0.944
var(Extrinsic motivation)	1					
var(Autonomous motivation)	1					
var(Prosocial motivation)	1					
cov(ε.High income*ε.Self-actualization)	0.171	0.087	1.97	0.049	0.001	0.341
cov(ε.High income*ε.Social change)	-0.099	0.046	-2.15	0.031	-0.190	-0.009
cov(ε.Job security*ε.Social change)	0.089	0.037	2.39	0.017	0.016	0.161
cov(ε.Helping others*ε.Social change)	0.286	0.045	6.39	0.000	0.198	0.374
cov(ε.Extrinsic motivation*ε.Autonomous motivation)	0.120	0.053	2.27	0.023	0.017	0.223
cov(ε.Extrinsic motivation*ε.Prosocial motivation)	-0.045	0.052	-0.88	0.380	-0.147	0.056
cov(ε.Autonomous motivation*ε.Prosocial motivation)	0.303	0.063	4.84	0.000	0.180	0.426

Notes: Table S1 presents the full SEM output for the confirmatory factor analysis without the hybrid item 'career options'. The latent factors (extrinsic, autonomous, prosocial motivation) are standardized (=1) so that the coefficients show correlations with the latent factors. Estimation method for the SEM was a Full-Information Maximum Likelihood (FIML). Robust standard errors are clustered on the subject-level for N=1,052 subjects. The total number of observations is 1,775. See Table 4 for goodness-of-fit statistics.

© 2026| Stefanos A. Tsikas (Tsikas.Stefanos@mh-hannover.de), Volkhard Fischer

B. Exploratory Factor Analysis (In which profession would you like to work later?)

Table S2: Exploratory factor analysis – Career aspirations

Variable	Factor1	Factor2	Factor3	Factor4	Factor5	Factor6	Uniqueness
University hospital	-0.253	0.055	**0.841**	0.056	-0.040	0.030	0.204
General hospital	-0.156	-0.039	0.206	0.053	**0.583**	-0.066	0.504
General practice	**0.990**	0.011	-0.133	0.010	0.000	-0.000	0.000
Gen. Practice (rural area)	**0.793**	-0.044	-0.133	0.010	-0.058	0.160	0.330
Specialized practice	0.280	0.065	-0.214	0.030	0.095	-0.185	0.832
Research & Development	-0.114	**0.422**	**0.472**	0.123	-0.279	-0.145	0.502
Pharmaceutical industry	-0.007	**0.902**	0.061	0.009	0.068	0.008	0.182
Public sector	0.149	**0.323**	-0.110	0.187	0.060	0.097	0.817
Development Aid	0.012	0.018	0.046	**0.999**	-0.000	-0.000	0.000
Military doctor	0.016	0.144	0.078	0.121	0.088	**0.372**	0.811
Variance	1.811	1.127	1.078	0.812	0.447	0.234	
Proportion	0.311	0.194	0.185	0.140	0.077	0.040	

Notes: Rotated factor loadings (extraction method: Maximum Likelihood principal factors; rotation: Varimax) of the ten items „In which profession would you like to work later?", rated on a scale from 1 = very unwillingly to 6 = very willingly. Six factors were retained. Number of parameters = 45. Number of observations = 2,154. LR test: $\chi^2 = 4{,}512.45$. $p<0.001$. Eigenvalues before factor rotation: Factor 1 = 1.958; Factor 2 = 1.091; Factor 3 = 1.278; Factor 4 = 0.812; Factor 5 = 0.447; Factor 6 = 0.233. Factor loadings > 0.32 are indicated in bold numbers. Standardized factor scores for the six factors retrieved after the factor analysis are used as predictors in Table 5 in the main text.

Based on the results of the EFA in Table S2, standardized factor scores for the following dimensions of desired professions were estimated and used for the fixed- and mixed effects regressions in Table 5 of the main text:

- Factor 1: General practice
- Factor 2: Research & Development
- Factor 3: University hospital
- Factor 4: Social work
- Factor 5: General hospital
- Factor 6: Military career

C. English translation of the HSM-questionnaire

Screenshots from the preview-function of the software evasys, with which the survey is conducted.

1. INTRODUCTION

With this survey on study motivation and study progress, we want to find out which aspects and experiences before and during your medical studies can have an impact on the success of your studies. In addition, your information should help us to continuously develop the curriculum and the study conditions at MHH. Many information are only required when you complete the questionnaire for the first time, other aspects are only asked about if you have already gained experience in studying medicine. At the end of the questionnaire, we ask for your consent to link your answers with data on your course of study. Please first indicate below which year of study you are currently in:

Your current academic year: [5 ▾]

<< Previous   Next >>

GENERAL INFORMATION

Why did you apply to study medicine at Hannover Medical School (multiple answers possible)?

- ☐ Proximity to the place of residence
- ☐ Reputation of the MHH
- ☐ Attractiveness of the university's city
- ☐ MHH model study programm (HannibaL)
- ☐ Other resasons

What is your current living situation?

- ○ Parents' home
- ○ Dorm
- ○ Shared flat
- ○ Own (rented) flat
- ○ Other

Where did you grow up? (multiple answers possible)

- ☐ rural area
- ☐ small town
- ☐ medium-sized town
- ☐ suburban area
- ☐ big city

Approximately how long does it take you to get from your accommodation to the MHH campus (in minutes)? [Please select... ▾]

<< Previous   Next >>

© 2026| Stefanos A. Tsikas (Tsikas.Stefanos@mh-hannover.de), Volkhard Fischer

HIGH SCHOOL INFORMATION

Please indicate here which advanced courses you took during your school years (multiple answers possible).

- [ ] Sciences
- [ ] Foreign languages
- [ ] History/Politics/Economics
- [ ] Latin/Ancient Greek
- [ ] Geography
- [ ] Maths/Physics
- [ ] Physical Education
- [ ] German studies
- [ ] Arts/Music
- [ ] Another course

In which of the following areas were you involved during your time at school? (multiple answers possible)

- [ ] Music
- [ ] Sports
- [ ] Culture/Arts
- [ ] Politics
- [ ] Natural sciences
- [ ] Other areas

School committee work / student work / representation (multiple answers possible)

- [ ] Class speaker (secondary level I)
- [ ] Class speaker (secondary level II)
- [ ] Class president ("Jahrgangssprecher"
- [ ] School spokesperson
- [ ] Student council
- [ ] Mediator
- [ ] Prefect
- [ ] Other
- [ ] None

Did you spend time abroad as part of an exchange programme during your school years? If yes, for how long?

- [ ] No
- [ ] < 1 month
- [ ] 1 - 4 months
- [ ] 4 - 8 months
- [ ] 9 - 12 months
- [ ] > 12 months

Did you do voluntary service after leaving school?

( ) Yes ( ) No

What extracurricular activities did you regularly take part in? (multiple answers possible)

- [ ] Recreational sports
- [ ] Competitive sports
- [ ] Amateur musician
- [ ] Active musician in, e.g., a choir or a band
- [ ] Youth counselling
- [ ] Volunteer work
- [ ] Other

Have you completed school internships in the medical field?

( ) Yes ( ) No

Have you completed voluntary internships in the medical field?

( ) Yes ( ) No

<< Previous

Next >>

CHOICE OF STUDY PROGRAMME

Before starting your studies, have you already...

...started or completed vocational training?	(●) yes - completed	( ) yes - started	( ) no

In which medical field did you start or complete your training (multiple answers possible)?

- [ ] Nursing care
- [ ] Administration
- [ ] Medical technical assistant
- [ ] Medical assistant
- [ ] Dental assistant
- [ ] Paramedics
- [ ] Prosthetic dentistry
- [ ] Other

...been employed? (●) yes ( ) no

Field of employment (multiple answers possible):

- [ ] In your trained profession
- [ ] Other
- [ ] Secondary and temporary jobs in the medical field
- [ ] Secondary and temporary jobs in the non-medical field

Were you already enrolled at another university before studying at MHH? (●) yes ( ) no

What was the focus of your previous degree programme (multiple answers possible)?

- [ ] Sciences
- [ ] Humanities
- [ ] Social sciences, economics
- [ ] Engineering
- [ ] Law
- [ ] Another focus

Did you graduate in this degree programme? ( ) yes (●) no

<< Previous Next >>

STUDY MOTIVATION I

What best characterises your situation before starting your studies? [Please select...]

How long have you wanted to study medicine? Please state the period of time until you started your studies. [Please select...]

How certain are you at the moment that medicine is your desired course of study? very certain ( ) ( ) ( ) ( ) ( ) ( ) not at all certain

How motivated are you currently to complete your studies? fully motivated ( ) ( ) ( ) ( ) ( ) ( ) not at all motivated

How has your motivation to study changed compared to last year? less motivated ( ) ( ) ( ) ( ) ( ) more motivated

<< Previous Next >>

© 2026| Stefanos A. Tsikas (Tsikas.Stefanos@mh-hannover.de), Volkhard Fischer

STUDY MOTIVATION II

Please rate what role the following reasons currently play in your choice of the study programme:

	big role					no role
To get a prestigious job	○	○	○	○	○	○
To contribute to social change	○	○	○	○	○	○
To obtain a secure professional position	○	○	○	○	○	○
To help other people	○	○	○	○	○	○
To achieve good income potential	○	○	○	○	○	○
Self-actualization	○	○	○	○	○	○
To have a lot of contact with other people	○	○	○	○	○	○
To pursue subject-specific interests	○	○	○	○	○	○
To have many career opportunities	○	○	○	○	○	○

<< Previous Next >>

STUDY MOTIVATION III

How confident are you that you will complete your studies?	completely certain	○	○	○	○	○	○	not at all certain
How important is it to you to complete your studies quickly (in the standard period of study)?	very important	○	○	○	○	○	○	not at all important

Here you can explain the reasons for your assessment of the standard period of study in more detail: [ ]

How important is it to you to complete your studies with a very good average grade?	very important	○	○	○	○	○	○	not at all important

Have you considered dropping out of your degree programme or changing university or subject during the course of your studies? ◉ yes - dropout ○ yes -transfer ○ no

For what reasons have you considered dropping out of your studies or changing university or subject? [ ]

At what point did you consider dropping out of your studies or changing subject? (multiple answers possible) ☐ Year 1 ☐ Year 2 ☐ Year 3 ☐ Year 4 ☐ Year 5

<< Previous Next >>

© 2026| Stefanos A. Tsikas (Tsikas.Stefanos@mh-hannover.de), Volkhard Fischer

STUDY PROGRESS

In terms of the workload during my studies, I am...	underwhelmed	○	○	○	○	○	○	overwhelmed
In terms of the level of demands in the degree programme, I am...	underwhelmed	○	○	○	○	○	○	overwhelmed

Have you started or already completed a doctoral thesis? ◉ yes - started ○ yes - completed ○ no

Are you doing or have you done your doctorate via StrucMed or KlinStrucMed? ○ yes ○ no

When did you start your doctorate? (please select month and year)

Month: Please select...

Year
(maximum 4 characters)

Did you study abroad while you were enrolled at the MHH? ◉ yes ○ no

Approximately how many months did you study abroad?

How many clinical traineeships have you completed so far?

<< Previous   Next >>

ASSESSMENT OF COMPETENCIES

To what extent do you have the following competences?

	very strong				none at all
... research specialised literature and sources independently	○	○	○	○	○
...to read specialised literature in English	○	○	○	○	○
...to write texts in English	○	○	○	○	○
... critically evaluating scientific developments in medicine	○	○	○	○	○
...to independently assess medical research results	○	○	○	○	○
...to integrate scientific findings into medical practice	○	○	○	○	○
... application of the principles of evidence-based medicine	○	○	○	○	○
... interpret statistical key figures independently	○	○	○	○	○
... write scientific texts independently	○	○	○	○	○
... present own research/research results orally	○	○	○	○	○

<< Previous   Next >>

© 2026| Stefanos A. Tsikas (Tsikas.Stefanos@mh-hannover.de), Volkhard Fischer

SELF-ASSESSMENT

To what extent do the following statements apply to you?

	not at all	rather not applicable	neither/nor	rather applicable	completely
I'm rather cautious, reserved	○	○	○	○	○
I trust others easily and believe in the good in people.	○	○	○	○	○
I tend to be lazy.	○	○	○	○	○
I am relaxed and don't let stress upset me.	○	○	○	○	○
I have very little artistic interests.	○	○	○	○	○
I come out of my shell, I'm sociable.	○	○	○	○	○
I tend to criticise others.	○	○	○	○	○
I complete tasks thoroughly.	○	○	○	○	○
I easily become nervous and insecure.	○	○	○	○	○
I am imaginative.	○	○	○	○	○

<< Previous Next >>

CAREER CHOICE

Which area would you like to work in later?

	very much					very reluctantly
In a university hospital	○	○	○	○	○	○
In a general hospital	○	○	○	○	○	○
In a practice as a general practicioner	○	○	○	○	○	○
In a general practice in a rural area	○	○	○	○	○	○
In a practice as a medical specialist	○	○	○	○	○	○
In academic research	○	○	○	○	○	○
In the pharmaceutical industry	○	○	○	○	○	○
In the (public) healthcare sector	○	○	○	○	○	○
In development aid	○	○	○	○	○	○
In the medical service of the military	○	○	○	○	○	○

<< Previous Next >>

© 2026| Stefanos A. Tsikas (Tsikas.Stefanos@mh-hannover.de), Volkhard Fischer

SOCIODEMOGRAPHIC INFORMATION

How do you mainly finance your studies? — Please select...

How many children do you have? — ○ none ○ 1 ○ 2 ○ 3 ○ >3

Is German your mother tongue? — ○ yes ○ no

How long have you been living in Germany? — ○ < 1 year ○ > 1 to 3 years ○ >3 to 5 years ○ > 5 years

Where was your father born? — ○ Germany ○ Foreign EU country ○ Non-EU country

Where was your mother born? — ○ Germany ○ Foreign EU country ○ Non-EU country

What is your parents' highest professional qualification?

Father — Doctorate

Mother — Degree from an university

In which specialisation did your father obtain his degree? — Please select...

In which field of study did your mother obtain her degree? — Please select...

<< Previous Next >>

DECLARATION OF CONSENT

I give my consent for my details to be combined with data on my study progress for quality assurance and statistical analyses. All information will be anonymised and not passed on to third parties. — ◉ yes ○ no

Thank you for your participation!

<< Previous

© 2026| Stefanos A. Tsikas (Tsikas.Stefanos@mh-hannover.de), Volkhard Fischer
