# Supplementary material for: Dimensions and temporal stability of medical students’ motivation: longitudinal evidence from a panel data survey
Source: BMC Med Educ. 2026 Aug 25;26:1367. doi: 10.1186/s12909-026-10249-z (PMC13504741; doi:10.1186/s12909-026-10249-z)
Supplement: Supplementary file 2 [file 12909_2026_10249_MOESM1_ESM.docx]

# **Appendix**

| **Table A:** Structural Equation Model (SEM), full output | | | | | | |
| --- | --- | --- | --- | --- | --- | --- |
| Factor | Coeff. | Robust  SE | z | P>z | 95%-confidence interval | |
| **Extrinsic motivation** |  |  |  |  |  |  |
| High income | 0.839 | 0.023 | 37.24 | 0.000 | 0.795 | 0.884 |
| Job security | 0.670 | 0.026 | 25.94 | 0.000 | 0.620 | 0.721 |
| Prestige | 0.642 | 0.023 | 27.73 | 0.000 | 0.597 | 0.687 |
| Career options | 0.235 | 0.038 | 6.12 | 0.000 | 0.159 | 0.310 |
|  |  |  |  |  |  |  |
| **Autonomous motivation** |  |  |  |  |  |  |
| Self-actualization | 0.760 | 0.056 | 13.51 | 0.000 | 0.650 | 0.870 |
| Academic interests | 0.430 | 0.045 | 9.56 | 0.000 | 0.341 | 0.518 |
| Career options | 0.255 | 0.046 | 5.58 | 0.000 | 0.165 | 0.344 |
|  |  |  |  |  |  |  |
| **Prosocial motivation** |  |  |  |  |  |  |
| Helping others | 0.564 | 0.058 | 9.81 | 0.000 | 0.451 | 0.677 |
| Social contacts | 0.944 | 0.089 | 10.58 | 0.000 | 0.769 | 1.119 |
| Social change | 0.487 | 0.135 | 3.62 | 0.000 | 0.223 | 0.751 |
|  |  |  |  |  |  |  |
| var(ε.High income) | 0.296 | 0.038 |  |  | 0.230 | 0.380 |
| var(ε.Job security) | 0.551 | 0.035 |  |  | 0.487 | 0.623 |
| var(ε.Prestige) | 0.588 | 0.030 |  |  | 0.532 | 0.649 |
| var(ε.Career options) | 0.859 | 0.025 |  |  | 0.811 | 0.909 |
| var(ε.Self-actualizazion) | 0.422 | 0.086 |  |  | 0.284 | 0.628 |
| var(ε.Academic interests) | 0.815 | 0.039 |  |  | 0.743 | 0.895 |
| var(ε.Helping others) | 0.682 | 0.065 |  |  | 0.566 | 0.822 |
| var(ε.Social contacts) | 0.109 | 0.168 |  |  | 0.005 | 2.233 |
| var(ε.Social change) | 0.763 | 0.131 |  |  | 0.544 | 1.068 |
| var(Extrinsic motivation) | 1 |  |  |  |  |  |
| var(Autonomous motivation) | 1 |  |  |  |  |  |
| var(Prosocial motivation) | 1 |  |  |  |  |  |
|  |  |  |  |  |  |  |
| cov(ε.High income*ε.Social change) | -0.133 | 0.046 | -2.91 | 0.004 | -0.223 | -0.044 |
| cov(ε.Job security*ε.Social change) | 0.090 | 0.037 | 2.44 | 0.015 | 0.018 | 0.162 |
| cov(ε.Helping others*ε.Social change) | 0.226 | 0.103 | 2.19 | 0.029 | 0.024 | 0.428 |
| cov(ε.Social contacts*ε.Social change) | -0.537 | 0.622 | -0.86 | 0.350 | -1.176 | 0.682 |
| cov(ε.Extrinsic motivation*ε.Autonomous motivation) | 0.178 | 0.048 | 3.71 | 0.000 | 0.084 | 0.273 |
| cov(ε.Extrinsic motivation*ε.Prosocial motivation) | -0.012 | 0.053 | -0.23 | 0.819 | -0.116 | 0.092 |
| cov(ε.Autonomous motivation*ε.Prosocial motivation) | 0.306 | 0.048 | 6.43 | 0.000 | 0.213 | 0.400 |
| Notes: Table A presents the full SEM output for the confirmatory factor analysis presented in Figure 1. The latent factors (extrinsic, autonomous, prosocial motivation) are standardized (=1) so that the coefficients show correlations with the latent factors. Estimation method for the SEM was a Full-Information Maximum Likelihood (FIML). Robust standard errors are clustered on the subject-level for N=1,218 subjects. The total number of observations is 2,193. | | | | | | |
